# The effects of major dietary patterns on patients with type 2 diabetes: Protocol for a systematic review and network meta-analysis

**DOI:** 10.1371/journal.pone.0306336

**Published:** 2024-06-28

**Authors:** Hongyu Chen, Yuanyuan Wang, Song Ge, Wanyang Li, Jing Li, Wei Chen

**Affiliations:** 1 School of Nursing, Chinese Academy of Medical Sciences & Peking Union Medical College, Peking Union Medical College Hospital, Beijing, China; 2 Department of Intensive Care Unit, The First Affiliated Hospital of Zhejiang Chinese Medical University (Zhejiang Provincial Hospital of Chinese Medicine), Hangzhou, China; 3 Department of Natural Sciences, University of Houston-Downtown, Houston, Texas, United States of America; 4 Department of Clinical Nutrition, Chinese Academy of Medical Sciences & Peking Union Medical College, Peking Union Medical College Hospital, Beijing, China; 5 School of Nursing, Chinese Academy of Medical Sciences & Peking Union Medical College, Beijing, China; Tehran University of Medical Sciences, ISLAMIC REPUBLIC OF IRAN

## Abstract

**Background:**

Type 2 diabetes mellitus (T2DM) represents a significant worldwide health issue, experiencing an increasing incidence rate. Effective dietary strategies are vital for T2DM management, but the optimal dietary patterns remain debated due to inconsistent research outcomes and single-outcome reporting. Network Meta-Analysis (NMA) provides a powerful approach for integrating data from randomized controlled trials (RCTs), enabling a detailed evaluation of the impact of different dietary patterns. This document presents our strategy for a systematic review and network meta-analysis, aimed at assessing the influence of key dietary patterns on glycemic control, lipid profiles, and weight management in individuals with Type 2 Diabetes Mellitus (T2DM).

**Materials and methods:**

Adhering to the Preferred Reporting Items for Systematic Reviews and Meta-Analysis Protocols (PRISMA-P) and network meta-analyses guidelines, we conducted a comprehensive search of PubMed, EMBASE, and the Cochrane Library, without language or date restrictions. Our objective is to assess the efficacy of various dietary interventions in managing Type 2 Diabetes Mellitus (T2DM). We used standardized mean differences for pairwise comparisons and a Bayesian framework for ranking interventions via Surface Under the Cumulative Ranking Curve (SUCRA). Key analyses include heterogeneity, transitivity, and sensitivity assessments, along with quality and risk evaluations using the Cochrane Collaboration’s tool and the Grading of Recommendations, Assessment, Development, and Evaluation (GRADE) system.

**Ethics and dissemination:**

This systematic review and network meta-analysis involve aggregate data from previous trials, obviating the need for additional ethical approval. The search strategy will be executed starting October 2023, with all searches completed by December 2023, to encompass the most current studies available. Findings will be shared through academic conferences and peer-reviewed journals focused on diabetes care and nutrition.

**Trial registration:**

PROSPERO registration number CRD42023465791.

## Introduction

The vast majority of diabetes diagnoses worldwide are classified as Type 2 diabetes mellitus (T2DM), representing more than 90% of total cases. T2DM results from insulin resistance and impaired insulin secretion [1] and has emerged as a significant global health challenge [2]. With over 536 million individuals affected globally as of 2021 [3], T2DM poses a significant health challenge, projected to impact 783 million by 2045. The disease contributes to severe health complications [4–6] and a considerable burden on healthcare systems [7].

Dietary management plays a crucial role in T2DM care [8], offering potential for significant improvements in glycemic control [9], lipid profiles [10], and weight management [11]. However, the optimal dietary pattern remains a subject of debate, largely due to the difficulty in comparing the effects of multiple dietary patterns directly [12].

Network meta-analysis (NMA) has gained prominence as a statistical technique [13–15], enabling the evaluation and comparison of multiple interventions’ effects [16,17]. However, previous NMA studies have yielded conflicting conclusions. For instance, one study highlighted the Mediterranean diet as the most effective for glycemic control [18], while another pointed to the ketogenic diet’s superiority [19]. Additionally, these studies often did not thoroughly examine other critical T2DM-related outcomes, such as changes in weight and waist circumference [10]. A notable study, published in 2017 [20], covered blood glucose control, lipid management, and weight reduction, but the emergence of numerous high-quality randomized controlled trials (RCTs) [21–25] since then has made these earlier syntheses less reflective of the current evidence. These contradictions, the focus on singular outcomes, and the increasing volume of recent RCTs highlight the urgent need for an updated, comprehensive synthesis of evidence.

This research seeks to comprehensively assess and evaluate the effects of predominant dietary patterns on the management of T2DM, incorporating both direct and indirect comparisons to provide healthcare professionals and patients with clear, evidence-based guidance on dietary choices.

## Methods

### Study design

This systematic review and network meta-analysis has been officially registered in the PROSPERO international registry for systematic reviews (Registration ID: CRD42023465791). Our approach will follow the standards set by the Preferred Reporting Items for Systematic Review and Meta-Analysis Protocols (PRISMA-P) [26] (S1 File) alongside the guidelines for Systematic Reviews and Network Meta-Analyses [27].

### Inclusion and exclusion criteria

#### Types of studies

We will exclusively include RCTs that investigate the effects of dietary patterns on the management of T2DM. Non-RCTs, observational studies, and reviews will be excluded, as they may not provide the level of evidence required for our analysis.

#### Types of participants

In this systematic review and network meta-analysis, we will include participants from primary studies who have been diagnosed with T2DM exclusively based on the diagnostic criteria provided by the American Diabetes Association (ADA), which includes fasting plasma glucose levels of 126 mg/dL (7.0 mmol/L) or higher, 2-hour plasma glucose levels of 200 mg/dL (11.1 mmol/L) or higher during an oral glucose tolerance test (OGTT), Hemoglobin A1c (HbA1c) levels of 6.5% (48 mmol/mol) or higher, or in patients with classic symptoms of hyperglycemia, a random plasma glucose of 200 mg/dL (11.1 mmol/L) or higher [28]. Studies involving individuals with other types of diabetes or comorbidities that significantly affect metabolic parameters will be excluded.

The comorbidities leading to exclusion include, but are not limited to:

- Severe renal impairment or renal failure, as these conditions can profoundly affect glucose and lipid metabolism.
- Advanced liver diseases such as cirrhosis, which can influence glucose production and storage.
- Congestive heart failure, where dietary restrictions and fluid management may impact nutritional interventions.
- Cancer, as cancer and its treatments can significantly alter body weight, metabolism, and nutritional status.
- Other specific endocrine disorders (excluding diabetes), such as untreated thyroid diseases, which can affect metabolic rates and glucose metabolism.

#### Types of interventions

We will include intervention trials that involving at least one of the following dietary regimens and an appropriate control.

An "appropriate control" in the context of our study is defined as:

- Standard Care Control: Groups receiving general dietary advice without specific emphasis on the dietary patterns under investigation.
- Placebo Control: For interventions where dietary supplements are involved, placebo controls that mimic the intervention in form but not in substance.
- No Intervention Control: Groups receiving no specific dietary advice or intervention during the study period.

Dietary Patterns Defined

The term ’dietary pattern’ encompasses the aggregate of foods and drinks that make up a person’s overall dietary consumption across a period. This systematic review and network meta-analysis aims to assess the efficacy of key dietary patterns in managing T2DM, including but not limited to:

- Low-carbohydrate diet: Defined as diets with less than 25% carbohydrate intake of total energy intake [29].
- Moderate-carbohydrate diet: Characterized by 25% to 45% carbohydrate intake of total energy intake [29].
- Low-fat diet: Defined by a fat contribution of <30% to total energy intake [29].
- Vegetarian diet: Exclusively excludes meat, poultry, and fish [30].
- High protein diet: Signified by proteins contributing >20% to total energy intake [31].
- Ketogenic diet: Prescribed as 5% to 10% carbohydrate intake of total energy intake [32].
- Mediterranean dietary pattern: Comprising elements such as fruits, vegetables, olive oil, legumes, cereals, fish, and modest consumption of red wine during meals [33].
- Dietary approach to stop hypertension (DASH): Emphasizing a high intake of fruits and vegetables [34].
- Low-glycemic index/load diet: Involving dietary regimens wherein the glycemic index and/or glycemic load values have been documented [35].
- Recommended diet: Following guidelines based on American Diabetes Association’s recommendations [36].

Intervention periods of at least three months will be included [10].

In our systematic review and network meta-analysis, we made the decision to exclude specific dietary regimens for the following reasons:

- Dietary Supplements or Single Foods: Studies focused solely on dietary supplements (e.g., vitamin C) or single foods (e.g., nuts) were excluded because our aim is to evaluate the impact of comprehensive dietary patterns on T2DM management, rather than the effects of isolated nutritional components.
- Dietary Supplements as Placebos: Similarly, studies using dietary supplements as placebos do not align with our objective to assess whole dietary patterns.
- Very Low-Energy Diets: Interventions utilizing extremely low-calorie diets (less than 600 kcal/day) were excluded due to their distinct metabolic effects and potential health risks, which may not be comparable to the broader dietary patterns under review [37].
- Medication as Placebos: Studies employing medication as placebos were excluded to maintain focus on dietary interventions without the confounding effects of pharmacological treatments.
- Co-interventions (Exercise or Medication): We excluded studies with co-interventions that were not consistently applied across all groups to isolate the effects of dietary patterns from those of other interventions [38].

#### Types of comparators

Studies comparing one dietary pattern against another or against a control group (e.g., usual diet) will be included.

#### Types of outcomes

Primary Outcomes:

- Blood Glucose Control:
  - Hemoglobin A1c (HbA1c) levels
  - Fasting Plasma Glucose (FPG)
  - 2-hour Postprandial Glucose (PPG)

Secondary Outcomes:

- Lipid Profiles:
  - Low-Density Lipoprotein Cholesterol (LDL-C)
  - High-Density Lipoprotein Cholesterol (HDL-C)
  - Total Cholesterol
  - Triglycerides
- Weight Reduction:
  - Body Mass Index (BMI)
  - Weight loss in kilograms or percentage of body weight lost
- Blood Pressure: Systolic and Diastolic Blood Pressure (SBP & DBP)
- Insulin Sensitivity: Homeostatic Model Assessment for Insulin Resistance (HOMA-IR)
- Inflammatory Markers: C-reactive protein (CRP), etc.

### Search strategy

For our systematic review and network meta-analysis, we plan to conduct an exhaustive search of PubMed, Embase, and the Cochrane Library, starting from their inception to the current date, without imposing any limits on publication dates. By adopting this strategy, we aim to capture the entirety of existing data regarding the efficacy of different dietary patterns in managing Type 2 Diabetes Mellitus (T2DM), ensuring a comprehensive overview of the evidence. The search strategy is designed to be inclusive, capturing all relevant RCTs published up to December 2023, providing a complete and up-to-date analysis of the literature.

The selection of search terms is derived from the study’s focus on participants, interventions, outcomes, and types of research. Our search strategy will incorporate both Medical Subject Headings (MeSH) and non-controlled vocabulary terms where suitable, including terms like ‘Diabetes Mellitus, Type 2’ and ‘Type 2 Diabetes’. To integrate these terms effectively, we will employ Boolean operators ’AND’ and ’OR’, and utilize search modifiers such as truncation, quotation marks for phrases, and wildcards to accommodate minor variations across different databases. The comprehensive search strategies tailored for three specific databases will be detailed in the S2 File.

To reduce the risk of publication bias, our search strategy will extend to locating unpublished studies via global trial registration sites such as the United States National Medical Clinical Trials Library and the World Health Organization International Clinical Trials Registry Platform, aiming to uncover active trials. Furthermore, a hand search will be conducted on the reference lists and bibliographies of all discovered articles, and we will scrutinize conference abstracts and journal publications for relevant data.

### Selection process

**Fig 1:**
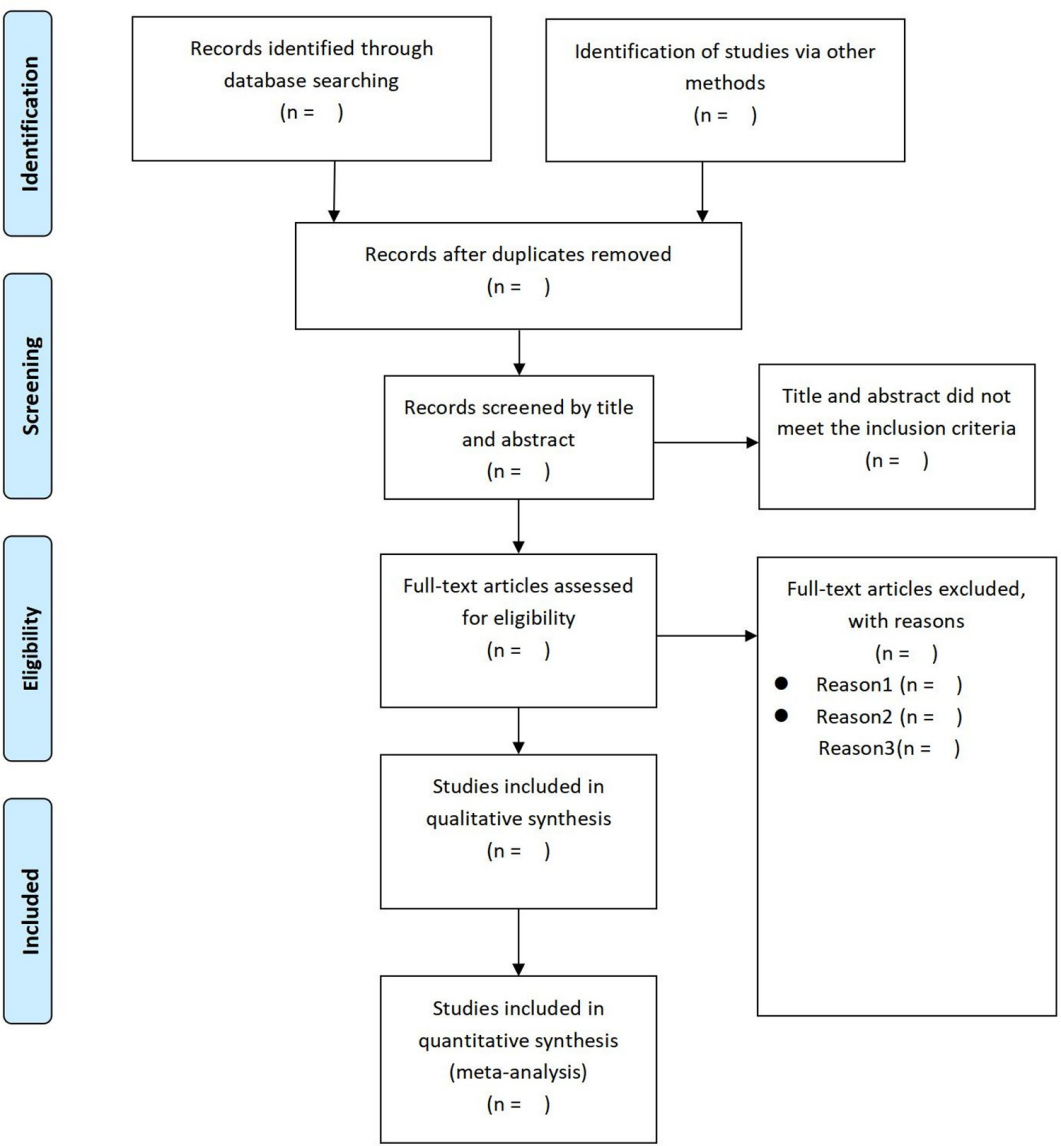
PRISMA flow diagram.

All collected articles will be uploaded to EndNote X9, which will assist in the elimination of duplicate entries. Following the removal of duplicates, two independent reviewers (CHY and WYY) will perform a preliminary examination of the titles and abstracts against the predefined inclusion criteria. Studies failing to match these criteria will be removed. Those qualifying in the preliminary phase will be subjected to an in-depth full-text assessment conducted independently by the same reviewers (CHY and WYY). Throughout the literature selection process, both reviewers will work independently. Differences or disagreements will be settled through dialogue and consultation to reach a mutual agreement. Should it become essential, an experienced third reviewer (CW) will independently assess the data to deliver a conclusive judgment. A graphical depiction of the study selection methodology will be presented, adhering to the PRISMA 2020 guidelines for the reporting of systematic reviews [39] (Fig 1).

### Data extraction

Data from the chosen studies will be extracted by two independent reviewers (CHY and WYY) utilizing a specifically designed data extraction form. This form includes study characteristics, participant demographics, details of dietary interventions, and relevant outcome measures. Discrepancies will be addressed by engaging in discussions and, if necessary, consulting with a third reviewer (CW) for resolution.

### Quality assessment

The assessment of quality and bias risk in the included RCTs will be conducted thoroughly utilizing the Cochrane Collaboration’s risk of bias tool [40]. This comprehensive assessment will be conducted independently by two reviewers (CHY and WYY) to ensure objectivity and reduce the potential for bias. The assessment will cover several domains, including randomization process, allocation concealment, blinding of participants and personnel, blinding of outcome assessment, incomplete outcome data, selective reporting, and other biases that may affect the validity of the study findings.

When differences arise among reviewers, we will attempt to achieve consensus via dialogue. Should a consensus remain elusive, a third reviewer (CW) will be called upon to render the decisive verdict. This multi-level review process is designed to enhance the reliability and credibility of our quality assessment. Furthermore, the results of the quality assessment will be summarized and presented in the form of a risk of bias graph and summary table, providing a transparent overview of the evidence base’s quality included in our review.

### Data synthesis and analysis

For pairwise meta-analysis, traditional meta-analytical methods will be utilized to directly contrast the impacts of varying dietary patterns. Since some outcome measures, such as blood glucose control, lipid management, and weight reduction, are typically reported as continuous variables with a consistent unit across studies, we will use the standardized mean difference (SMD) as the effect measure. We will calculate these effect measures, encompassing numerical values and averages, and determine the 95% confidence intervals (CI) for the Standard Mean Difference (SMD). A p-value below 0.05 will be deemed to indicate statistical significance.

In conducting our network meta-analysis, the Bayesian approach will be adopted to juxtapose the impacts of various dietary patterns. Intervention rankings will be determined utilizing the Surface Under the Cumulative Ranking Curve (SUCRA) methodology. For the execution of the network meta-analysis, the "BUGSnet" package within R software [41] will be our tool of choice. The outcomes from both direct and indirect analyses will be visually depicted through a network diagram [42].

Additionally, sensitivity analyses will be performed to evaluate the solidity of our research outcomes. This will involve the exclusion of studies identified as having a significant bias risk or those that fail to comply with the intention-to-treat principle. Moreover, should the analysis encompass a considerable number of studies (more than 10), we will employ funnel plots and statistical methods to investigate any potential publication bias [43].

### Heterogeneity assessment

Before combining the effect size in our meta-analysis, we will assess heterogeneity among included studies based on participant characteristics, interventions, and outcomes. We will use the χ2 test (Cochrane Q) and the inconsistency index (I^2^) to evaluate heterogeneity of the included studies. An I^2^ value exceeding 50% will be indicative of substantial heterogeneity. In response to such heterogeneity, we will opt for the random-effects model to conduct the meta-analysis. This choice acknowledges and appropriately addresses the variability among the included studies, ensuring a more conservative and reliable synthesis of the data. Conversely, if I^2^ is less than 50%, we will use the fixed-effects model for the combination of effect sizes [44].

### Assumption of transitivity

The principle of transitivity holds paramount importance in network meta-analysis. For our study, we will take into account variables such as the age of patients, duration of symptoms, severity of disease, method of treatment, type of intervention, as well as its duration and intensity, as potential effect modifiers, drawing from a review of existing literature. It will be presumed that these key effect modifiers remain consistent across all studies included in our analysis. Should there be any concern regarding a breach of transitivity, we will delve into examining any effect modifiers that could potentially influence the outcomes of interventions.

### Inconsistency assessment in the network

Maintaining consistency is crucial for network meta-analysis. The evaluation of discrepancies between direct and indirect comparisons will be carried out through the application of Node-splitting analysis. This technique segregates the data pertaining to a specific comparison of interventions into direct and indirect categories, thereby facilitating an examination of any variances.

### Evidence quality assessment

The evaluation of evidence quality will be conducted utilizing the Grading of Recommendations, Assessment, Development, and Evaluation (GRADE) approach [45]. Evidence will be classified into four categories: very low, low, moderate, or high, taking into consideration factors such as bias risk, evidence indirectness, variability, accuracy, and the potential for publication bias [46].

### Patient and public involvement

Patients and the public did not have direct involvement in the conduct of this systematic review, as it primarily involved data analysis of previously conducted clinical trials.

## Discussion

The escalating global incidence of T2DM underscores the urgency in identifying effective management strategies [2], with dietary patterns playing a pivotal role [47]. Despite extensive research, the debate regarding optimal dietary management persists, largely due to the variability in study outcomes and the predominance of research focusing on singular dietary effects.

Previous meta-analyses have significantly advanced our understanding of the efficacy of various dietary patterns in managing Type 2 Diabetes Mellitus (T2DM). For instance, research by Schwingshackl et al. highlighted the benefits of the Mediterranean diet [18], while Jing et al. focused on the advantages of ketogenic diets [19]. However, these studies often concentrated on specific outcomes such as glycemic control, which provides a somewhat limited view of dietary impacts. Many of these analyses did not encompass a comprehensive range of metabolic outcomes, which are crucial for fully understanding the multifaceted effects of dietary patterns on T2DM management. This gap in the literature suggests a need for a more holistic approach to dietary analysis. Our planned systematic review and network meta-analysis aims to fill this gap by providing a broad evaluation of dietary patterns that considers not only glycemic control but also lipid profiles, weight management, and other metabolic markers. By integrating these diverse outcomes, our study will offer a more complete picture of how different diets affect various aspects of health in individuals with T2DM.

Our findings are anticipated to have significant implications for clinical practice. For instance, by identifying the most effective dietary patterns [48], healthcare professionals can tailor nutritional advice more precisely, which may improve patient adherence and outcomes. Moreover, this evidence-based approach could be used to update existing guidelines on dietary management for T2DM, ensuring that they reflect the latest research.

In conclusion, our research offers a significant advancement in understanding and managing T2DM through dietary interventions. By mapping out the effectiveness of various dietary patterns and situating them within the current research landscape, we furnish stakeholders with the evidence needed to forge targeted and effective dietary strategies.

However, it is crucial to acknowledge potential limitations inherent in our approach. Our study’s reliance on RCTs for network meta-analysis may introduce variability due to differences in study design, populations, and interventions. Despite our comprehensive analysis, the generalizability of findings might be limited by the diversity of dietary patterns and T2DM severities across studies. Additionally, publication bias and the availability of data could affect the robustness of our conclusions. These factors underscore the need for cautious interpretation of our results and their application in clinical and public health settings.

### Ethics and dissemination

#### Ethics

Given that this study constitutes a systematic review and network meta-analysis based on aggregated data from existing trials, it does not necessitate further ethical approval.

#### Dissemination

The findings of this study will be shared at both national and international levels. We aim to present our results at relevant conferences focusing on nutrition, diabetes management, and systematic reviews. Furthermore, we intend to publish our research in peer-reviewed journals specializing in diabetes care and nutrition. To enhance the reach and impact of our findings, we are contemplating hosting informative events or webinars tailored for individuals with T2DM and healthcare professionals.

## Supporting information

S1 Checklist Research checklist—PRISMA-P (Preferred Reporting Items for Systematic review and Meta-Analysis Protocols) 2015 checklist. (DOCX)

S1 File Research checklist—PRISMA-P (Preferred Reporting Items for Systematic review and Meta-Analysis Protocols) 2015 checklist. (DOCX)

S2 File The detailed search strategies for three databases. (DOCX)
