# Supplementary material for: The effects of major dietary patterns on patients with type 2 diabetes: Protocol for a systematic review and network meta-analysis
Source: PLoS One. 2024 Jun 28;19(6):e0306336. doi: 10.1371/journal.pone.0306336 (PMC11213329; doi:10.1371/journal.pone.0306336)
Supplement: S2 File — (DOCX) [file pone.0306336.s003.docx]

**Searching Strategy**

Database: PubMed

| Search No | Indexed and Keyword Terms |
| --- | --- |
| 1  2  3  4  5  6  7 | ((“Diabetes Mellitus, Type 2"[Mesh]))  ((Type 2 Diabetes[Title/Abstract]) OR (IDDM[Title/Abstract])) OR (NIDDM[Title/Abstract])) OR (MODY[Title/Abstract])) OR  (T2DM[Title/Abstract])) OR (Diabetes Mellitus, Noninsulin-Dependent[Title/Abstract]) OR (T2D[Title/Abstract]) OR  (Diabetes Mellitus, Ketosis-Resistant[Title/Abstract])) OR (Diabetes Mellitus, Ketosis Resistant[Title/Abstract]) OR (Ketosis-Resistant Diabetes Mellitus[Title/Abstract])  #1 OR #2  ((“Diet, Healthy"[Mesh]))  ((dietary patterns[Title/Abstract]) OR "portfolio diet"[Title/Abstract] OR "Mediterranean diet"[Title/Abstract] OR "MedDiet"[Title/Abstract] OR "MeDiet"[Title/Abstract] OR "High-Carbohydrate diet"[Title/Abstract] OR "high protein diet*"[Title/Abstract] OR "Diet, Fat-Restricted"[Mesh] OR (("low fat" OR "fat free" OR "fat-restricted" OR "fat restriction" OR "restricted fat") AND diet*[Title/Abstract]) OR (("ketogenic diet" OR "Ketosis" OR "low carb*") AND diet*[Title/Abstract]) OR "atkins diet"[Title/Abstract] OR "modified atkins diet"[Title/Abstract] OR "high-protein low-carbohydrate diet"[Title/Abstract] OR "very low carbohydrate diet"[Title/Abstract] OR "very low-carbohydrate diet"[Title/Abstract] OR "low CHO diet"[Title/Abstract] OR "carbohydrate restricted diet"[Title/Abstract] OR "CHO restricted diet"[Title/Abstract]) OR low[Title/Abstract] AND (diet*[Title/Abstract] AND ("gi"[Title/Abstract] OR "glycemic  index"[Title/Abstract] OR "glycemic load"[Title/Abstract] OR "glycemic indices"[Title/Abstract]))  #4 OR #5  #3 AND #6  Filters: Randomized Controlled Trial, Clinical Trial, Clinical Trial, Phase I, Clinical Trial, Phase II, Clinical Trial, Phase III, Clinical Trial, Phase IV |

Database: Embase

| Search No | Indexed and Keyword Terms |
| --- | --- |
| 1  2  3 | ('Diabetes Mellitus, Type 2'/exp) OR (Type 2 Diabetes or IDDM or NIDDM or MODY or T2DM or Diabetes Mellitus, Noninsulin-Dependent or T2D or Diabetes Mellitus, Ketosis-Resistant or Diabetes Mellitus, Ketosis Resistant or Ketosis-Resistant Diabetes Mellitus):ti,ab  ('Diet, Healthy'/exp) OR ('dietary patterns':ti,ab OR 'portfolio diet':ti,ab OR 'Mediterranean diet':ti,ab OR 'MedDiet':ti,ab OR 'MeDiet':ti,ab OR 'High-Carbohydrate diet':ti,ab OR 'high protein diet*':ti,ab OR 'Diet, Fat-Restricted'/exp OR (('low fat' OR 'fat free' OR 'fat-restricted' OR 'fat restriction' OR 'restricted fat'):ti,ab AND diet*:ti,ab) OR (('ketogenic diet' OR 'Ketosis' OR 'low carb*'):ti,ab AND diet*:ti,ab) OR 'atkins diet':ti,ab OR 'modified atkins diet':ti,ab OR 'high-protein low-carbohydrate diet':ti,ab OR 'very low carbohydrate diet':ti,ab OR 'very low-carbohydrate diet':ti,ab OR 'low CHO diet*':ti,ab OR 'carbohydrate restricted diet*':ti,ab OR 'CHO restricted diet*':ti,ab OR low:ti,ab AND (diet*:ti,ab AND ('gi' OR 'glycemic index' OR 'glycemic load' OR 'glycemic indices'):ti,ab)).  ('randomized controlled trial'/exp OR 'randomized controlled trial':ti,ab OR 'clinical trial':ti,ab OR 'controlled clinical trial':ti,ab  #1 AND #2 AND #3 |

Database: Cochrane Library (CENTRAL)

| Search No | Indexed and Keyword Terms |
| --- | --- |
| 1  2  3  4  5  6  7 | MeSH descriptor: [Diabetes Mellitus, Type 2] explode all trees  (Type 2 Diabetes or IDDM or NIDDM or MODY or T2DM or Diabetes Mellitus, Noninsulin-Dependent or T2D or Diabetes Mellitus, Ketosis-Resistant or Diabetes Mellitus, Ketosis Resistant or Ketosis-Resistant Diabetes Mellitus):ti,ab,kw    #1 OR #2  MeSH descriptor: [Diet, Healthy] explode all trees  ('dietary patterns' or 'portfolio diet' or 'Mediterranean diet' or 'MedDiet' or 'MeDiet' or 'High-Carbohydrate diet' or 'high protein diet' or 'Diet, Fat-Restricted' or (('low fat' or 'fat free' or 'fat-restricted' or 'fat restriction' or 'restricted fat') and diet) or (('ketogenic diet' or 'Ketosis' or 'low carb*') and diet*) or 'atkins diet' or 'modified atkins diet' or 'high-protein low-carbohydrate diet' or 'very low carbohydrate diet' or 'very low-carbohydrate diet' or 'low CHO diet' or 'carbohydrate restricted diet' or 'CHO restricted diet*') or ('low' and (diet* and ('gi' or 'glycemic index' or 'glycemic load' or 'glycemic indices'))):ti,ab,kw  #4 OR #5  #3 AND #6 |
